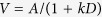# Undervaluing delayed rewards explains adolescents’ impulsivity in inter-temporal choice: an ERP study

**DOI:** 10.1038/srep42631

**Published:** 2017-02-15

**Authors:** Yunyun Huang, Ping Hu, Xueting Li

**Affiliations:** 1 Department of Psychology, Renmin University of China, Beijing 100875, China

## Abstract

Adolescence has frequently been characterized as a period of choice impulsivity relative to adulthood. According to the control-integrated valuation model of inter-temporal choice, this choice impulsivity may be driven partly by an age-related difference in reward processing. We hypothesized that, compared to adults, adolescents would undervalue delayed rewards during reward processing and would thus be more impulsive in inter-temporal choice. To test this hypothesis at the behavioural and neural levels, we first measured the choice impulsivity of 18 adolescents and 19 adults using a delay discounting task (DDT). Then, we recorded event-related potentials (ERPs) from the participants while they were performing the valuation task, in which monetary gains and losses were either immediate or delayed. The behavioural results showed that adolescents were more impulsive than adults in the DDT. The ERP results showed that, whilst both groups valued immediate rewards, implied by a similarly strong feedback-related negativity (FRN) effect associated with immediate outcomes, adolescents devalued delayed rewards more than adults did, as they produced a significantly smaller FRN effect associated with delayed outcomes. As predicted, the mediation analysis revealed that the adolescents’ lower FRN effect of delayed outcomes underpinned their stronger impulsive decision making in the DDT.

*“I would that there were no age between ten and three-and-twenty, or that youth would sleep out the rest, for there is nothing in between but getting wenches with child, wronging the ancientry, stealing, fighting…”*

*— Shakespeare, The Winter’s Tale, Act III, Scene 3*

The above soliloquy from Shakespeare describes the youth of his day. It still rings familiar currently. Indeed, adolescents have long been associated with heightened impulsivity as exemplified by drug use^1^,^2^, unintentional injuries (especially car accidents)^3^, and unprotected sexual activity^4^. Hall (1904) included these as part of his view of adolescent storm and stress, agreeing that “a period of semi-criminality is normal for all healthy adolescents“^5^,^6^. The increased impulsivity across adolescence highlights the need to better understand the psychological and neural processes underlying these changes. In the present experiment, first, we compared adolescents and adults to test their behavioural differences in terms of choice impulsivity and then to test the neurocognitive effects that might impact the behavioural differences.

Choice impulsivity (CI) refers to making impulsive decisions in choosing smaller-sooner (SS) over larger-later (LL) rewards^7^. Various reviews have found that CI is moderately correlated with trait impulsivity^8^, related to difficulties in delaying immediate gratification or exerting self-control^9^, and can be reliably measured by inter-temporal choice tasks^7^. For these and other reasons, CI has been recommended for inclusion in impulsivity research. The delay discounting task (DDT), an inter-temporal choice paradigm, has been found to be an effective tool for quantifying choice impulsivity^10^,^11^. In the DDT, participants make successive choices between receiving an SS reward or an LL reward. Often, the SS reward is chosen over the LL reward. Such a preference for SS rewards can be understood in terms of delay discounting; that is, the subjective valuation of rewards declines as a function of time^12^,^13^. Numerous studies have shown that the discount rates decline from adolescence to adulthood^14^,^15^. However, the precise mechanisms underpinning this developmental change remain poorly understood.

The more impulsive behaviour of adolescents in the DDT might be attributed to their aberrant reward processing and/or poor capability of cognitive control^10^. According to the control-integrated valuation account, reward processing and cognitive control are two core processes during inter-temporal choice^16^,^17^,^18^, the underlying neural systems of which mature at different rates across adolescence. Specifically, there is a relatively early development of the valuation system associated with reward processing, followed by a slower maturation of the control system associated with cognitive control during adolescence^19^,^20^,^21^. Therefore, the adolescents who are more prone to choose the immediate rewards compared to adults might have a relatively low subjective value of the delayed reward, and/or they might have especially poor cognitive control.

Previous studies on this issue have found that weak cognitive control, rather than age-related differences of reward processing, uniquely accounted for the impulsivity of adolescents in inter-temporal choice^10^,^22^,^23^. However, as these studies measured the reward processing in the DDT, in which participants are faced with two choices, an SS reward and a LL reward, the valuation process and choice process cannot be dissociated from one another. Adult research attempting to separate the valuation process and choice process of the DDT would inform our understanding of this issue.

The cumulative evidence in adults has indicated that reward processing and its underlying neuronal substrates contribute substantially to the impulsivity of adults, but their precise contribution remains controversial. Some studies have shown that undervaluation of delayed monetary rewards leads to the impulsivity of adults^24^,^25^. Another study, however, has shown that overvaluation of immediate rewards instead of undervaluation of delayed rewards was associated with more impulsive behaviour in adults^26^. As the latter study used an unappealing delayed reward (25 cents in 6 months) that equated to a loss for all participants, the finding is questionable. On balance, the studies tended to indicate that undervaluation of delayed rewards contributed to adult impulsivity, which led us to expect the same in adolescents’ choice impulsivity.

Choice impulsivity research based on the feedback-related negativity (FRN), an event-related potential (ERP) component, is useful for the present purpose of exploring the neurocognitive mechanisms that may affect choice impulsivity. The FRN has been suggested as a suitable measure for assessing the developmental trajectory of reward evaluation in ERP studies^27^,^28^. The FRN is a negative deflection peaking approximately 200–300 ms post-stimulus, which was more pronounced for negative events (e.g., a negative feedback or an outcome that is worse than expected) than for positive events^29^,^30^,^31^,^32^. Recent studies suggested that the FRN may be better thought of as reward positivity that is increased for positive events^33^. Developmental research on the FRN or reward positivity suggested that the basic neural mechanism for reward evaluation was in place and adult-like by adolescence^28^,^34^,^35^.

Thus, there is support from the existent literature for the use of theFRN to measure reward evaluation in adolescents and adults. If adolescents’ higher choice impulsivity relative to adults is affected by their undervaluation of delayed rewards, the FRN would vary between the two age groups. In the present experiment, we were particularly interested in FRN as a neural measure to investigate whether age differences in reward evaluation lead to increased impulsivity in adolescents on a DDT and a choice-independent valuation task. Electroencephalograms (EEGs) were recorded while participants were performing the valuation task, in which monetary gains and losses were either immediate or delayed. Prior to the valuation task, participants also completed the DDT without recording EEGs. We hypothesized that compared to adults, adolescents would make more impulsive choices in the DDT, as revealed by a higher discount rate (Hypothesis 1), and they would undervalue delayed rewards, as indicated by a decreased FRN effect in response to delayed outcomes in the valuation task (Hypothesis 2). Finally, adolescents’ greater undervaluation of delayed outcomes would impact the effect of age groups on their higher choice impulsivity (Hypothesis 3).

## Results

### Overview

The results are presented in three parts in accordance with the three hypotheses. First, the main effect of the age groups on choice impulsivity was tested by analysing the DDT choices, and the results supported Hypothesis 1: adolescents were more impulsive than adults, as shown by their higher discount rates. Second, the reward evaluation difference between adolescents and adults was tested by analysing the dFRN data, and these results were consistent with Hypothesis 2 in showing a smaller FRN effect of delayed outcomes in adolescents than in adults. Third, mediation analysis based on the overall data was conducted, and the results showed that devaluation of delayed outcomes impacted the effect of age groups on adolescents’ higher choice impulsivity, as predicted by Hypothesis 3.

### Hypothesis 1: Difference in choice impulsivity between adolescents and adults

Choice impulsivity behaviour was expressed in terms of the discount rate (*k*) and log transformed into log *k* for analysis. A larger log *k* in absolute value indicated a stronger preference for SS over LL rewards. An independent sample *t*-test of the transformed discount rates revealed that the discount rate of adolescents (*M*_*adolescents*_ = −1.336 ± 0.139) was significantly higher than that of adults (*M*_*adults*_ = −1.724 ± 0.486), which was in support of Hypothesis 1 (*t*(35) = −3.339, *p* = 0.003).

### Hypothesis 2: Reward evaluation difference between adolescents and adults

**Figure 1:**
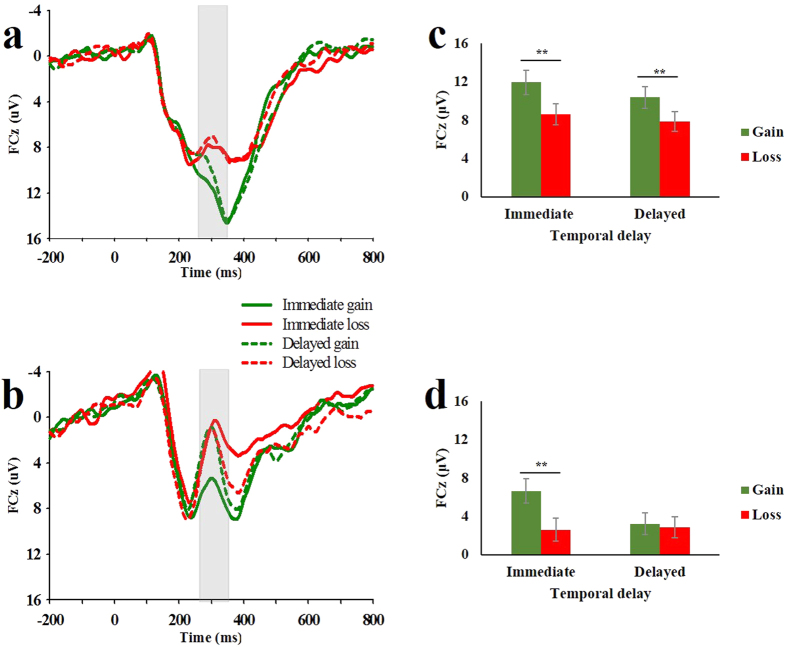
The grand-averaged ERPs. The FRN amplitudes at FCz after the presentation of reward feedback for adults (**a**) and adolescents (**b**) are shown. Shaded areas indicate the time range during which the FRN was evaluated (250–350 ms). Bar charts provide mean FRN amplitudes across each condition corresponding to the grand-averaged FRN for adults (**c**) and adolescents (**d**). Error bars represent the within-group standard errors (SEs), *p < 0.05, **p < 0.01.

**Figure 2:**
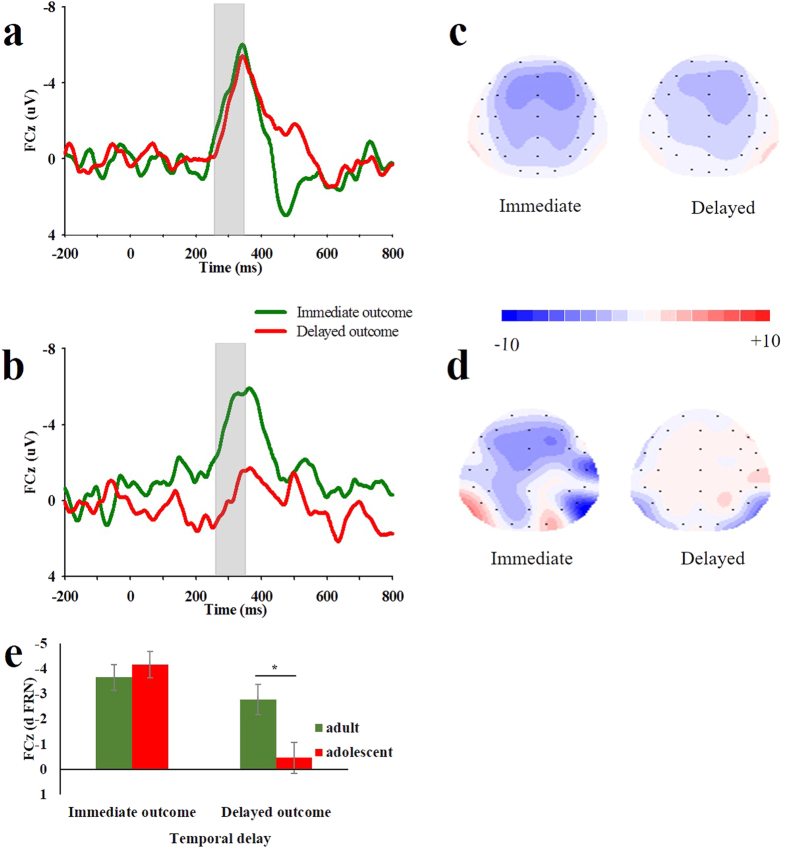
The difference waveforms and scalp topographies. Difference waveforms of adults (**a**) and adolescents (**b**) at the FCz site are shown. Shaded areas indicate the time range during which the dFRN was evaluated (250–350 ms). The maps for adults (**c**) and adolescents (**d**) in the immediate condition and delayed condition are shown. (**e**) Bar charts provide mean dFRN amplitudes elicited by immediate and delayed rewards for adults and adolescents. Error bars indicate the within-group standard errors (SEs), *p < 0.05.

Figure 1 illustrates the grand-averaged ERPs recorded at channel FCz for adults and adolescents, and Fig. 2 shows the immediate and delayed difference waves (dFRN) and their associated scalp distributions. To test Hypothesis 2, we focused on the dFRN and conducted a two-way repeated-measures ANOVA comprising the factors of temporal delay (immediate vs. delayed) and age groups (adolescents vs. adults). The results revealed a significant main effect of temporal delay (*F*(1,35) = 18.582, *p* < 0.001, *η*^2^ = 0.347), showing a larger FRN effect (more negative dFRN) in the immediate condition compared to the delayed condition. The main effect of age groups was not significant (*F*(1,35) = 2.291, *p* = 0.139, *η*^2^ = 0.061).

Importantly, the interaction between temporal delay and age groups was significant (*F*(1,35) = 6.992, *p* = .012, *η*^2^ = 0.167). A subsequent simple effect analysis showed that the dFRN in adolescents was not significantly different from adults in the immediate condition (F(1,35) = 0.480, *p* = 0.492), whilst both groups could distinguish between gain and loss in the immediate condition (*M*_*adolescents*_ = −4.170 μV, *t*_(17)_ = −6.658, *p* < 0.001; *M*_*adults*_ = −3.666 μV, *t*_(18)_ = −9.477, *p* < 0.001). In contrast, adults produced a significantly larger FRN effect than adolescents did in the delayed condition (F(1,35) = 7.11, *p* = 0.012). Specifically, while adults evaluated delayed gain significantly more than delayed loss (*M*_*adults*_ = −2.776 μV, *t*_(18)_ = −5.563, *p* < 0.001), adolescents evaluated delayed gain as equal to delayed loss (*M*_*adolescents*_ = −0.463 μV, *t*_(17)_ = −0.643, *p* = 0.529). These results suggested that age difference in reward evaluation was primarily due to the dFRN elicited by delayed outcomes; that is, adolescents undervalued delayed outcomes relative to adults, as hypothesized.

### Hypothesis 3: Mediation effect of reward evaluation

To assess whether age-related changes in delay discounting were mediated by concomitant changes in the dFRN of the delayed outcomes, the correlation coefficients between group, discount rate (log *k*), and the dFRN of delayed outcomes were calculated. As the significant age group differences in the discount rate and dFRN of delayed outcomes have already been reported above, only the correlation between discount rate and the dFRN of delayed outcomes would be necessary here. The discount rate was significantly correlated with the dFRN of delayed outcomes (*r* = 0.451, *p* = 0.005) in the hypothesized direction; that is, higher discount rates in the DDT (higher choice impulsivity) were related to a smaller FRN effect of delayed outcomes (undervaluation of delayed outcomes).

**Figure 3:**
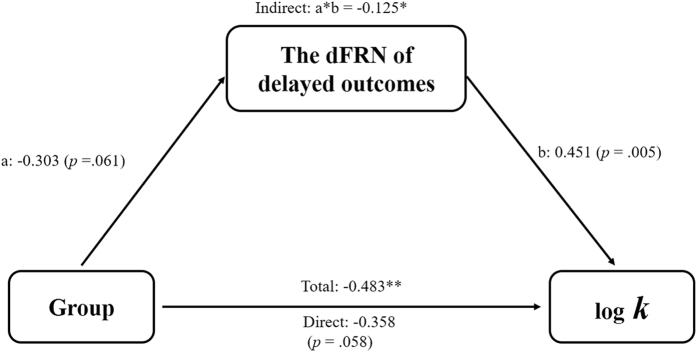
The mediation model for the effects of group on discount rate via the dFRN. Values are standardized regression coefficients, *p < 0.05; **p < 0.01.

In the mediation analysis, age groups were treated as an independent variable, the transformed discount rate (log *k*) as the outcome variable, and the dFRN of the delayed outcome as the mediator (see Fig. 3). The total effect of age groups on the discount rate was significant (total effect −0.483, *p* = 0.002). The mediation effect of FRN difference waves of delayed outcomes, as indicated by the indirect effect, was significant (indirect effect −0.125, SE = 0.067, 95% confidence intervals: −0.296, −0.039). After the indirect path was included in the model, the direct effect of age groups remained marginal significant (direct effect −0.358, *p* = 0.058). The overall results indicated partial mediation. Taken together, these results demonstrated that age-related changes in choice impulsivity could be partly explained by adolescents’ devaluation of delayed outcomes.

## Discussion

Although a wealth of research on inter-temporal choice has shown that adolescents are more inclined to opt for SS over LL rewards relative to adults, it remains to be determined whether this choice impulsivity is due to age-related differences in reward evaluation. To investigate this issue, we measured choice impulsivity of adolescents and adults in the DDT and measured their reward evaluation in the valuation task. At the behavioural level, adolescents were more impulsive than adults. At the neural level, both groups showed a similarly strong FRN effect in response to immediate outcomes, whilst adolescents produced a significantly smaller FRN effect in response to delayed outcomes. As a smaller FRN indicates undervaluation, the results showed that adolescents undervalued delayed outcomes relative to adults, and both age groups valued immediate outcomes equally. The pattern of contrasting FRN effects pinpointed the age-related neural differences in the devaluation of delayed outcomes. As predicted, the mediation analysis showed that the dFRN of delayed outcomes significantly mediated the behavioural impact of age groups on adolescents’ more impulsive decision-making. Overall, the age-related reward evaluation differences and the mediation effect of the dFRN of delayed outcomes indicated that the impulsivity of adolescents was partly driven by their devaluation for delayed outcomes subserved by the neural system underlying reward processing.

The discussion below relates our main findings to the existing literature, pointing out consistencies, discussing apparent discrepancies, and citing the limitations of the present study. The present finding of a greater FRN effect in the immediate condition than in the delayed condition, which indicates that both age groups value immediate outcomes more than delayed outcomes, is consistent with the common account of classic delay discounting. In this, the immediate reward serves as the reference point for future rewards, and the subjective value of future rewards would decrease with an increase in delay time^36^,^37^. Thus, the subjective value of gain-loss difference in the immediate condition is larger than that in the delayed condition, and both age groups in the present study value immediate outcomes. However, compared with adults, adolescents with a higher discount rate evidently place less value on delayed gains and decrease the subjective value of gain-loss difference in the delayed condition. Therefore, adolescents in the present study have stronger devaluation for delayed outcomes, as indicated by a significantly smaller FRN effect in response to delayed outcomes relative to adults. The above interpretation is also supported by neural evidence, which showed that the subjective value of delayed rewards was tracked by reward processing regions of the brain, and the activity in these regions decreased with delays in future rewards^24^,^38^. Further support is evident from study that has reported that more impulsive individuals had relatively decreased ventral striatal activity to delayed rewards^25^.

Our result that both adolescents and adults valued immediate outcomes equally, whereas adolescents devalued delayed outcomes relative to adults, suggests that adolescents have greater valuation differences between immediate and delayed outcomes compared to adults. This result is consistent with the study by Schmidt *et al*. that found more impulsive individuals showed bigger delayed-immediate outcome differences^39^. Adolescents’ low preference for delayed outcomes is in line with previous literature which indicated that adolescents were less inclined to plan ahead or anticipate the future consequences of their actions before acting^2^. This relatively weaker future orientation mainly lies in adolescents’ early development of the valuation system juxtaposed with the protracted maturation of the cognitive control system^40^,^41^.

However, the present finding that undervaluation of delayed outcomes caused adolescents’ choice impulsivity is inconsistent with Cherniawsky and Holroyd, who found that more impulsive adults overvalued immediate outcomes rather than undervalued delayed outcomes^26^. The discrepancy between the two studies may be the result of employing different reward magnitudes and temporal delays. The present study used a more appealing delayed reward with a large magnitude and a short delay (about US $1.5 in a month) in the valuation task. Its result, adults valued delayed rewards more than non-rewards, is consistent with our previous research^42^. Nevertheless, Cherniawsky and Holroyd used less appealing delayed rewards (25 cents in 6 months) in their study, and both high and low temporal discounters valued delayed rewards as equalling to non-rewards. We suspect that the converging results would have been observed for delayed outcomes if the same reward magnitude and temporal delay were used in both studies. In addition, we did not find support for the notion that adolescents’ heightened sensitivity to immediate outcomes drives their choice impulsivity. The absence of this immediacy effect may be due to the low level of affective content of the stimuli used in the study^10^. There is evidence that adolescents are particularly sensitive to social context, and that the presence of peers specifically increased their temporal discount rates^43^,^44^ and neural activity in the ventral striatum^45^. We expect that the pattern of overvaluing immediate outcomes in adolescents would be more likely to be found in the social context, an issue that awaits further investigation.

In the current study, we only found a partial mediation of reward evaluation. There is room left for other factors such as cognitive control. For example, van den Bos *et al*. have reported that developmental improvements in cognitive control from 8 to 25 years uniquely accounted for age-related changes in delay discounting^10^. Although this finding might have been hampered by the procedure of measuring reward evaluation in the DDT (which would have confounded the reward evaluation with reward choice), a recent study that assessed the valuation process and choice process separately has found that the slow development of cognitive control in children (6–13 y) accounted for their choice impulsivity^46^. Based on previous findings and our present results, reward processing and cognitive control may both contribute to the developmental changes in choice impulsivity. In line with this prediction, a recent longitudinal study has shown that the developmental path between the striatum (involved in reward processing) and the prefrontal cortex (involved in cognitive control) was an important underlying mechanism for the ability to delay gratification^47^.

Some issues arose from this study that should be addressed in future research. First, even though the use of valuation task with one reward presented at a time allows one to measure reward evaluation independent of the potential confounding factor of reward choice, there are differences between the valuation task and DDT other than choices being made or not, such as the presentation of one versus two reward options. This inconsistency in turn might have an impact on the relation between reward evaluation and choice impulsivity. Second, although our study implied that age-related differences in reward evaluation led to the choice impulsivity of adolescents, as the choice impulsivity and reward evaluation were measured separately by using different tasks, more evidence is still needed to make a rigorous causal inference. Future longitudinal research or studies that measure choice impulsivity and reward evaluation in the same task (i.e., the DDT, but with a separation of the valuation process and choice process) could further our understanding of how reward evaluation impacts adolescents’ choice impulsivity. Finally, our current results of discount rates in the DDT are based on the payment method of one random selected trial. Since both adolescents and adults were paid out according to the pay one method, the potential influences of payment method on delay discounting could be balanced between the two groups. However, as a recent study indicated that participants made riskier decisions with pay all than with pay one^48^, it is possible that the absolute values of discount rates might be influenced by the pay one method. Future research should take into account the possible influences of the payment method on the discount rates.

In summary, the main finding from the present study is that greater delay discounting in adolescents versus adults (measured by the DDT) is mediated by a reduced sensitivity to delayed outcomes in adolescents, as shown by the failure to show a difference in FRN amplitude to delayed gains versus losses. This finding has important implications for the neurocognitive mechanisms mediating choice impulsivity in adolescence. The mechanisms may have their roots in the underdeveloped reward neural system, which subserves the capacity for reward processing and outcome evaluation.

## Method

### Participants

The experiment participants were 21 middle school students and 21 students at a near-by university. The data from five participants were discarded before further analysis, due to excessive artefacts in the EEG data or an inadequate number of artefact-free ERP trials. The final study cohort consisted of 18 (8 males) adolescents (*M* = 14.72; *SD* = 0.90; range 14–16 years), and 19 (9 males) adults (*M* = 20.21; *SD* = 1.27; range 18–23 years). All participants provided written informed consent prior to the experiment. The experiment was conducted in accordance with the ethical guidelines of the American Psychological Association and was approved by the Renmin University of China Review Board.

### Stimuli and procedures

Participants completed a behavioural and a valuation session. In the behavioural session, participants completed the DDT to provide the choice impulsivity (indicated by discount rate) data. In the next session, which immediately followed, participants completed the valuation task, during which EEGs were recorded to provide the reward evaluation (indicated by the FRN) data. Participants were tested individually, one at a time.

Each participant completed the DDT of 78 inter-temporal choices, which were determined by a staircase procedure using E-Prime 2.0. In each choice, a SS reward paired with a LL reward were presented, from which the participant could choose. The SS reward was 10 RMB (about US $1.5), which the participant would receive immediately after the experiment. This amount was fixed in all 78 choices. The LL reward was designed to combine different sizes of reward and delay periods. Specifically, reward size varied from 10.5 to 32 RMB in 13 steps of 5%, 15%, 25%, 35%, 50%, 70%, 85%, 95%, 125%, 150%, 180%, 200% and 220% above the SS. The delay period (*D*) was 1 week, 2 weeks, or 1 month. Thus, there were 39 distinct LL rewards, each of which was paired with the SS to provide 39 pairs of choice. Each pair was repeated once to give a total of 78 inter-temporal trials. In each trial, participants chose between the SS and one of the 39 LLs. A hyperbolic discount function was fitted to the choice behaviour data of each participant to determine the individual discount rate (*k*).


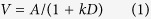


In this function, *V* was the amount in RMB of the SS reward, *A* was the amount in RMB of the LL reward, and *D* was the delay period in days (that is, 7, 14 or 30 days). A larger *k* would indicate a steeper discounting of the future reward; that is, a stronger preference for the SS reward over the paired LL reward. It would provide a behavioural measure of the participant’s choice impulsivity in the following manner: the larger the *k*, the stronger the participant’s choice impulsivity. A logarithmic transformation was applied to *k* to correct for an extreme positive skew in accordance with common practice in DDT data analysis^49^.

Both adolescents and adults conducted the same choice-independent valuation task^43^, coded in E-Prime 2.0, in which monetary gains and losses were either immediate or delayed. Hence, there were four experimental conditions, as follows: gain 10 RMB now, lose 10 RMB now, gain 10 RMB a month later, and lose 10 RMB a month later. Prior to the start of the task, participants were instructed to adopt any strategy to maximize their rewards by choosing between the two pictures. Unbeknownst to the participants, different types of feedback were predetermined and presented in equivalent numbers, regardless of their actual choice.

**Figure 4:**
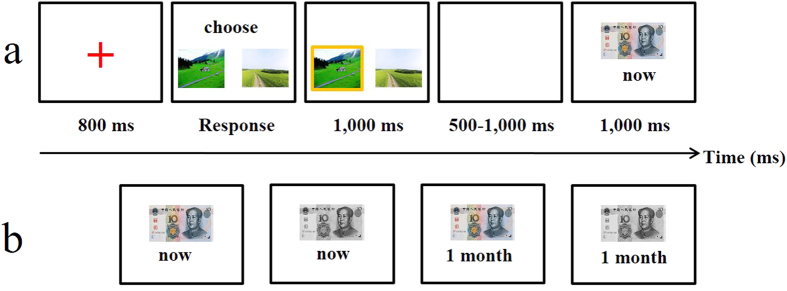
The valuation task procedure and feedback types. (**a**) The time course of stimulus presentation in the valuation task is shown. When two pictures (taken by Huang and cited from our previous study^37^) were presented, participants needed to make a choice between them by pressing the corresponding button. After 500–1,000 ms, a choice-independent feedback image was shown for 1,000 ms. (**b**) There were four types of feedback: gain 10 RMB now, lose 10 RMB now, gain 10 RMB a month later and lose 10 RMB a month later. Eq. (1) **Discount function**. *V* is the amount in RMB of the SS reward, *A* is the amount in RMB of the LL reward, *D* is the delay period in days of the LL reward, *k* is the discount rate, and a larger *k* indicates steeper discounting of the future reward.

The valuation task consisted of four blocks of 50 trials each (200 trials in total). As illustrated in Fig. 4a, for each trial, participants were presented with a choice between two pictures (taken by Huang and used for maintaining participant engagement) by pressing corresponding keys (F for the left picture and J for the right picture). The pictures remained on the screen until a choice was made. The chosen picture was highlighted by a yellow border for 1,000 ms. Then, participants waited for 500–1,000 ms, after which time a feedback image was displayed for 1,000 ms. Each feedback image consisted of a photo of the 10 RMB (the coloured photo indicates gain and the black-and-white photo indicates loss), with the time of the reward delivery written below as “Today” or “1 month” (Fig. 4b). After each block, a self-timed rest period was provided for participants.

Participants were informed that their final payment depended on the combination of their choice in the DDT and their performance on the valuation task. In the DDT, a participant’s choice on a trial would be randomly picked and delivered at the corresponding delay (today, 1 week, 2 weeks, or 1 month). In the valuation task, participants would receive 40 RMB (about US $6) as basic payment, and the additional monetary reward would be given according to the feedback of four randomly picked trials. The immediate rewards were distributed after participants had completed the experiment, and the delayed rewards were sent to them one month later.

### ERP recordings and analyses

EEGs were recorded from 40 scalp sites using a Quik-Cap with sintered Ag/AgCl electrodes (NeuroScan Inc., USA) according to the NuAmps International 10–20 System. The vertical electrooculograms (VEOGs) were recorded from electrodes placed above and below the left eye. The horizontal electrooculograms (HEOGs) were recorded from electrodes placed at the outer canthi of both eyes. The impedance of all electrodes was below 5 kΩ. The EEGs and EOGs were amplified (bandpass 0.1–100 Hz) and continuously sampled at 1000 Hz/channel for offline analysis. All the EEG data were referenced online to the left mastoid and re-referenced algebraically offline to the average of the left and right mastoids. In the offline analysis, ocular artefacts were corrected with an eye-movement correction algorithm. EEG epochs of 1,000 ms, with 200 ms of pre-feedback stimulus baseline, were extracted from the continuous data file for analysis. Trials containing excessive artifacts were excluded using a threshold of ± 90 μV. The EEG data were low-pass filtered using a 30 Hz low-pass (24 dB/Octave), and baseline-corrected by subtracting from each sample the average activity of that electrode during the baseline period.

The difference wave approach has been suggested as a good way to minimize component overlap when comparing ERPs across different age groups in previous research^28^. In the present study, we were interested in the age-related differences in reward evaluation in both the immediate and the delayed conditions. Therefore, we followed the methods of Lukie *et al*. and created two difference waves by subtracting the ERPs elicited by gains from ERPs associated with losses^28^,^50^. The difference-based FRN (dFRN) amplitude was measured as the mean amplitude of the difference wave within a 250–350 ms window post-stimulus. Based on visual inspection of the waveforms and its topographical distributions (Figs 1 and 2), 4-midline electrode sites (Fz, FCz, Cz and CPz) were initially selected for statistical analyses of the dFRN. Because the FRN effect at FCz was the largest, we focused on the FCz for further analyses.

The participant’s relative reward evaluation was indexed as the dFRN. If gain and loss did not differentially affect the ERP with a given condition, then the amplitude of the dFRN would be equal to zero. Otherwise, the amplitude of the dFRN would be significantly different from zero. A larger dFRN (more negative amplitude) indicates greater discrimination between gain and loss and higher motivational significance of the current condition. The dFRN data were analysed according to a 2 (temporal delay: immediate vs. delayed) × 2 (age groups: adolescents vs. adults) analysis of variance (ANOVA). Greenhouse-Geisser correction was used when the assumption of sphericity was violated. Effect sizes are provided as partial eta squared (*η*^2^).

Finally, to assess if the changes in delay discounting between age groups was mediated by concomitant changes in the dFRN of immediate and delayed outcomes, we first calculated the correlations among groups, the transformed discount rate (log *k*), the dFRN of immediate outcomes, and the dFRN of delayed outcomes. Then, we used the Marsbar toolbox^51^ to perform the mediation analysis.

## Additional Information

**How to cite this article:** Huang, Y. *et al*. Undervaluing delayed rewards explains adolescents’ impulsivity in inter-temporal choice: an ERP study. *Sci. Rep.* **7**, 42631; doi: 10.1038/srep42631 (2017).

**Publisher's note:** Springer Nature remains neutral with regard to jurisdictional claims in published maps and institutional affiliations.